# Acupuncture for erectile dysfunction in post-stroke patients: Study Protocol Clinical Trial (SPIRIT Compliant)

**DOI:** 10.1097/MD.0000000000019718

**Published:** 2020-04-10

**Authors:** Yanfeng Li, Xudong Yu, Ruijia Liu, Jisheng Wang, Sheng Deng, Bowen Liu, Chongyang Zhang, Haisong Li

**Affiliations:** a Department of Urology, Dongzhimen Hospital, Beijing University of Chinese Medicine, Beijing; b Graduate School of Beijing University of Chinese Medicine, Chaoyang; c Department of Andrology; d Department of Neurology; e Department of Acupuncture, Dongzhimen Hospital, Beijing University of Chinese Medicine, Beijing, China.

**Keywords:** acupuncture, post-stroke erectile dysfunction, protocol, randomized controlled trial

## Abstract

**Introduction:**

Erectile dysfunction refers to the continued inability of the penis to reach and maintain sufficient erections to achieve a satisfactory sex life and last at least 6 months. As part of traditional Chinese medicine, acupuncture has been widely used in clinical practice. In order to evaluate, the exact effect of acupuncture on the clinical efficacy of patients with Post-stroke Erectile dysfunction (PSED), this experiment uses randomized controlled experiments.

**Methods/design:**

This pragmatic randomized controlled trial will recruit 103 patients who are diagnosed with PSED. Simple randomization to conventional treatment with a 1:1 allocation ratio will be used. Ten 30-min acupuncture sessions will be provided to patients assigned to the Intervention group. All participants will continue to receive conventional treatment. The selection of outcomes will be evaluated by International Erectile Function Index-5 (IIEF-5) score at week 8.

**Discussion:**

This trial may provide evidence regarding the clinical effectiveness, safety, and cost-effectiveness of acupuncture for patients with PSED.

**Trial registration:**

ClinicalTrials.gov, ChiCTR2000030231, Registered on February 25, 2020.

## 1 Introduction

Erectile dysfunction (ED) refers to the continued inability of the penis to reach and maintain sufficient erections to achieve a satisfactory sex life and last at least 6 months.^[1]^ It is one of the most common types of male sexual dysfunction. It is also a common and frequently occurring disease in andrology, and it is increasing year by year.^[2]^ Based on the epidemiological studies of ED at home and abroad, it can be found that from the early 1990s to 2010, the prevalence of ED in men over 40 years of age has increased significantly.^[3]^ Although most surveys show that the prevalence of ED has a significant positive correlation with age, the majority of ED patients who come to andrology clinics are 30 to 60 years old. At the same time, it can be seen that long-term smoking and drinking, as well as cardiovascular diseases (CVD), endocrine diseases (diabetes and hyperlipidemia), etc have become high-risk factors affecting the incidence of ED, and the proportion has gradually increased. In contrast, men under 60 still have a strong need for sex and are willing to actively seek the help of a doctor.^[4]^ In contrast, the proportion of organic ED has increased significantly, has become the main type of ED, and has gradually moved forward with the age of high-risk factors. The age of the first occurrence of ED also continues to advance, and the course of disease continues to prolong.^5^,^6^ This has caused the problem that male erectile function and sexual needs of this age group are difficult to match, which seriously affects the quality of life of patients.

Previous studies have shown that ED and many CVD have common risk factors. Risk factors for CVD, such as age, body mass index, cholesterol, triglyceride smoking, hypertension, and smoking, were significantly correlated with ED. In addition, diabetes increases the risk of both, and endothelial dysfunction and arteriosclerosis are common features of both.^[7]^ Autonomic hyperfunction and altered hormone levels may be more complex mechanisms. The close correlation between CVD and cerebrovascular disease in terms of pathogenesis, pathophysiology, and prognosis has been recognized by the medical community.^[8]^ Therefore, it is reasonable to believe that ED is similarly related to cerebrovascular disease. With the change of people's life rhythm and eating habits, many major diseases that affect people's physical and mental health, such as CVD, digestive system diseases and malignant tumors, have a tendency to be younger, which is a hot and difficult point in clinical research. Stroke, also known as cerebrovascular accident, is a cerebrovascular disease with common neurological deficits and brain tissue damage caused by various inducing factors.^[9]^ The disease is characterized by high disability, high mortality and recurrence. Stroke, as an acute cerebrovascular disease, is the leading cause of death and disability in urban and rural residents. Previous studies on stroke have mainly focused on stroke mechanisms, high-risk factors, and treatment, and there have been fewer studies on sexual life dysfunction in patients with stroke. And young and middle-aged male stroke patients often have very high requirements for quality of life; patients are ashamed to open their teeth when they have secondary dysfunction, and the attention of medical staff and related knowledge reserves are relatively small, which seriously affects the treatment of patients. Acupuncture is safe, economical, accurate, convenient to operate, and has small side effects.^[10]^ It has unique advantages in limb dysfunction after stroke. Studies have shown that acupuncture therapy can stimulate the body's internal organs and immune functions, and can adjust physical skills in multiple forms, functions, and levels.

Based on the current needs of patients with Post-stroke Erectile dysfunction (PSED) that do not respond well to conventional treatments, and the need for clinical research on the level of evidence for efficacy, it is worthwhile to further explore the treatment model of acupuncture for PSED. Therefore, we will evaluate the clinical effectiveness and safety of acupuncture in the diagnosis and treatment of PSED. We hope that the results of this study will provide clinicians with acupuncture treatment for PSED and more treatment options.

## 2 Methods/design

### 2.1 Study design and settings

**Figure 1:**
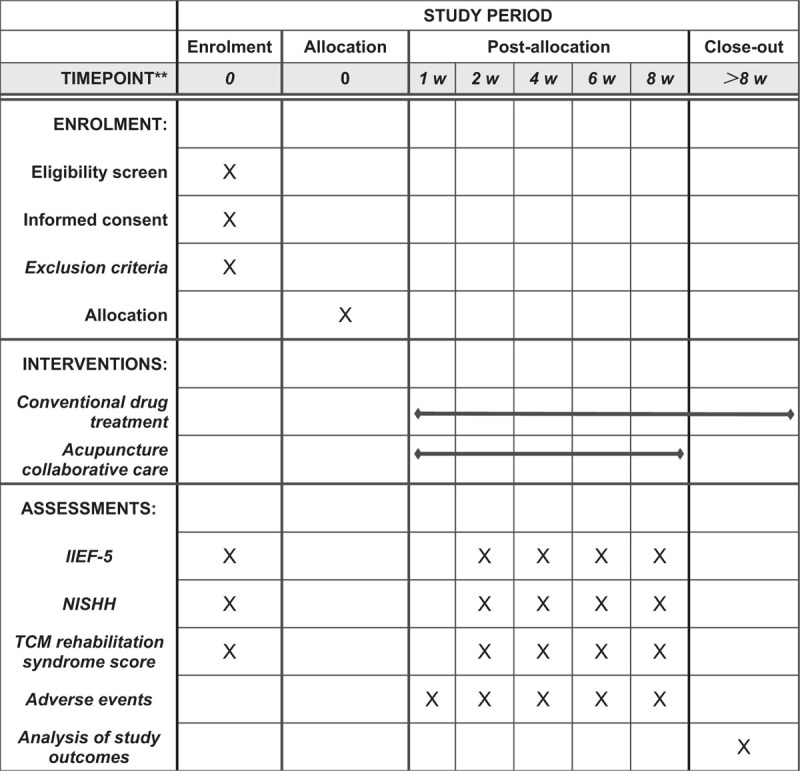
SPIRIT figure for the schedule of enrollment, interventions, and assessments.

This study will be a single-blinded, randomized controlled trial with 2 parallel groups. It will be conducted at the Dongzhimen Hospital Affiliated to Beijing University of Chinese Medicine, Beijing, China. This protocol was written and based on Standard Protocol Items: Recommendations for Interventional Trials guidelines. The participants will be informed about the research, procedures, risks, and benefits. If they agree, they will sign an informed consent form. Only those participants who read and agree to the protocol and who sign the informed consent form will take part of the study, following the schedule described in Figure 1.

### 2.2 Participants

**Figure 2:**
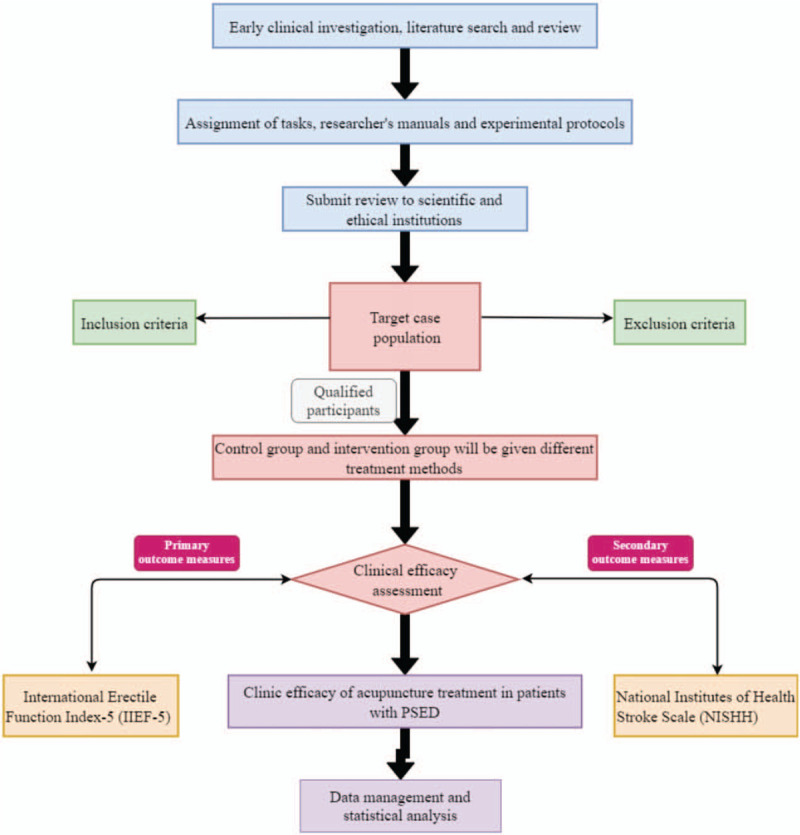
Study design flow chart.

The selected cases will be both outpatients and inpatients at Dongzhimen Hospital Affiliated to Beijing University of Chinese Medicine (Beijing, China). The diagnostic criteria refer to the “*Urological Surgery* · *Interstitial Bladder Inflammation Diagnostic Standard*” (Wu Jieping, Gu Fangliu, Guo Yinglu, Beijing Shandong Science and Technology Press) (Fig. 2).

#### 2.2.1 Diagnostic criteria

Western medicine diagnosis refers to “Chinese Urological Disease Diagnosis and Treatment Guide” and “Urinary Surgery” in ED-related diagnostic standards. The diagnosis of TCM refers to the “Guiding Principles for Clinical Research of New Chinese Medicines,” and the syndrome is identified as damp-heat-stasis syndrome type. Western medicine standards refer to the “Guide to the Diagnosis and Treatment of Integrated Chinese and Western Medicine of Chinese Cerebral Infarction” by the Chinese Society of Integrated Traditional Chinese and Western Medicine. Traditional Chinese Medicine refers to the “Stroke Diagnosis and Efficacy Evaluation Standards” formulated by the Chinese Medicine Administration's Encephalopathy Acute Scientific Research Collaboration.

#### 2.2.2 Inclusion criteria

This study will be conducted in China. Patients will be recruited from urology/neurology departments of Dongzhimen Hospital Affiliated to Beijing University of Chinese Medicine. We will enroll participants based on the following inclusion criteria:

1. Those who meet the western medical diagnostic criteria for impotence (ED) and occur after stroke;
2. 20 ≤ age ≤ 60 years old male, normal sexual desire;
3. The vital signs are stable and the stroke recovers;
4. All are first stroke.
5. Accept and sign the informed consent

#### 2.2.3 Exclusion criteria

Patients will be excluded if they meet the following criteria:

1. Sexual dysfunction exists before the patient's stroke;
2. Stroke patients with vascular malformations (arteriovenous fistula, arteriovenous malformations, aneurysms) and subarachnoid hemorrhage;
3. People with neurological diseases and nervous system infections;
4. Complicated sexually transmitted diseases;
5. People with severe liver and kidney damage;
6. Malignant tumor.

#### 2.2.4 Conditions for participants to suspend and withdraw from the clinical trial

Researchers participating in clinical trials should carefully record the reasons for the suspension of the trial and the relationship with the clinical trial. It is necessary to clearly record the unwillingness of the subjects to continue the clinical trials, put forward the reasons for withdrawing from the clinical trials, and record the evaluation indicators at the time of discontinuation in detail.

1. Those who cannot adhere to treatment;
2. Allergic reactions or serious adverse reactions during the test, the test should be suspended:
3. Those who have not been treated strictly according to the plan;
4. People who withdrew from the study on their own

### 2.3 Interventions

Control group: The control group will be given conventional treatment to regulate blood pressure, blood glucose, and blood lipid. At the same time, drugs such as nutritional nerves, promoting blood circulation, and removing blood stasis are used, adjusted according to the needs of the disease, and routine rehabilitation training is performed.

Intervention group: This group of patients will be given acupuncture combined with rehabilitation training. TCM acupuncture therapy: Acupoints include *Quchi*, *Waiguan*, *Hegu*, *Dicang*, *Sanyinjiao*, and *Zusanli*. The twisting technique will be used, leaving the needles for 30 min, and treated once a day for 2 weeks. Rehabilitation training is first of all passive activities. With the help of ropes, sticks and other tools, fists, leg lifts, cross fingers, etc are performed 3 times a day for 30 min each time. When the muscle strength returns to level 3, take active training, including voluntary turning, sitting exercises, standing exercises, walking training, etc. The total application time will be 8 weeks. At the same time, closely monitor the change of the condition in order to control the deterioration of the condition in time.

### 2.4 Outcome measures

#### 2.4.1 Primary outcome measures

We will use the International Erectile Function Index-5 (IIEF-5) as the main outcome indicator.

1. Recovery: IIEF-5 score ≥22 points after treatment
2. Significant effect: IIEF-5 score < 22 points after treatment, points improved ≥60%;
3. Effective: IIEF-5 points <22 points after treatment. The improvement of points is between 30% and 60%;
4. Ineffective: IIEF-5 points after treatment are <22 points, and the improvement of points is <30%. Total effective rate = (healed + markedly + effective)/total number of cases × 100%.

#### 2.4.2 Secondary outcome measures

We will use the National Institutes of Health Stroke Scale (NISHH) before and after treatment to assess patients’ daily living ability, including eating, transferring, grooming, bathing, toileting, walking, dressing, going down stairs, and urination control. Out of 100 points, the higher the score and the stronger the living ability. Observe the clinical efficacy, the judgment criteria are as follows: obvious effect: nausea, drowsiness, limb numbness, and other symptoms disappear, ADL score increased by more than 75%; improvement: clinical symptoms reduced, ADL score increased by 25% to 75%; invalid: clinical symptoms did not improve significantly The ADL score improved by <25%.

### 2.5 Sample size calculation

According to the clinical experience, the effective rate of PSED in the intervention group was *P*1 = 0.80. The PSED effective rate of the control group was *P*2 = 0.50 (([two-sided type I error rate of 0.05, α = 0.05]; [one-sided type I error rate of 0.10, β = 0.10]). Substituting *f* (α, β) into the formula is as follows: n1 = n2 = 10.5 × (0.8 × 0.2 + 0.50 × 0.35) ÷ 0.152 = 48. The lost follow-up rate of patients will be controlled at 10%, so an additional 5 cases are added to each group. n1 = n2 = 53, that is, 53 cases will be taken from each of the intervention group and the control group. According to Cohen, this effect size is considered “moderate.”

### 2.6 Randomization and blinding

Participants will be randomly allocated to the 2 groups through a sequence of numbers generated by a computer program before starting the selection process. The group assigned to each patient will be kept in a sealed envelope with the objective of concealing the assignment to the researcher, who will decide on the entry of subjects to the study. Given the nature of the interventions, the physiotherapists, and the patients, blinding will not be possible. However, the evaluator and statistician will be blinded to which group the subjects evaluated will belong.

### 2.7 Statistical analysis

Data management uses EXCEL software to build a database, double entry, check for outstanding values, and lock. Statistical analysis will be performed using SPSS 25.0 software for statistical analysis. The normality of the measurement data is tested. The data obeying the normal distribution is Student's *t* test, which is expressed by mean ± standard deviation. The data not obeying the normal distribution is rank sum test. And marginal homogeneity test; count data are expressed by rate and composition ratio, and comparison is performed by chi-square test; repeated measurement data are expressed by mean ± standard deviation, intra-group comparison is performed by analysis of variance of repeated measurement data, and inter-group comparison is by multivariate analysis of variance (MANOVA). *P* ≤ .05 indicates that the difference is statistically significant.

### 2.8 Data management

Information obtained from the evaluation of each participant will be recorded on a paper print-out. The information will then be handwritten on a paper document case report form and entered into an Excel file for future statistical analyses. In accordance with the Personal Information Protection Act, the names of all participants will not be disclosed, and a unique identifier number given during the trial will be used to identify participants. All of the participants will be informed that the clinical data obtained in the trial will be stored in a computer and will be handled with confidentiality. The participants’ written consent will be stored by the principal investigator.

### 2.9 Ethics

The study will be conducted under the Declaration of Helsinki principles, as well as following the norms of good clinical practice. Recruitment of patients has not started in this study. The study plan will be submitted to the ethics committee of the Dongzhimen Hospital Affiliated to Beijing University of Chinese Medicine for review. The study protocol will be approved by the ethics committee of the Dongzhimen Hospital Affiliated to Beijing University of Chinese Medicine. We will not start recruiting participants without the consent of the ethics committee. The protocol of this study has been registered in the Chinese Clinical Trial Registry with the number ChiCTR2000030231.

## 3 Discussion

The mechanism of ED occurs in many aspects, including blood vessels, nerves, endocrine, systemic diseases, local penile diseases, nutrition, psychological factors, and has a relationship with drugs and drugs. Penile erection is a hemodynamic process involving relaxation of the corpus cavernosum and its related arteriolar smooth muscle.^[11]^ Therefore, physiologically, the basic conditions of penile erection include a complete nerve conduction pathway, a sound penile tissue structure, and sufficient arterial filling pressure.^[12]^ All three are indispensable. The nerves that govern the penis are the autonomic and somatic nerves. The sympathetic and parasympathetic nerves in the cavernous body can regulate blood flow in the cavernous body when the penis is erect and weak. During sexual stimulation, parasympathetic nerves, non-adrenergic and non-cholinergic nerve endings and vascular endothelial cells release nitric oxide (NO) under the action of nitric oxide synthase (NOS). NO activates the guanylate cyclase of smooth muscle cells, which turns guanylate (GMP) into cyclic guanylate (cGMP). Increasing cGMP concentration in cells decreased Ca^2+^ concentration in cytoplasm.^13^,^14^ The smooth muscles of the penis relax, the blood flow in the penis increases, and the internal pressure of the cavernous body increases until the cavernous venous occlusion function starts, and the penis begins to erect and maintains ejaculation.^[15]^ As cGMP was degraded by smooth muscle cells type 5 phosphodiesterase (PDE-5), the penis turned into a weak state.

The factors that affect the sexual function of stroke patients are many and complex. Stroke location, area, disease occurrence and development process may affect patients’ sexual function. Therefore, this study made strict rules on case selection, excluding patients who could not take care of themselves and had emotional cognition disorders; when selecting cases, patients had a recent stroke >3 months to ensure that the stroke was stable and chose to comply as much as possible Patients with better sexuality and cooperation should conduct a questionnaire study to reduce the impact of other factors on the patient's sexual function.^[16]^ The manifestation of sexual dysfunction in young and middle-aged male patients is mainly ED. In sexual activity, men are often active acquirers, and the occurrence of ED is determined by multiple factors. Studies have shown that the high-risk factors that affect men's erections are mainly related to patients’ smoking history, high blood pressure, worries about recurrence of stroke, and daily antihypertensive receptor inhibitors. The possible reasons are:

1. the influence of subjective assessment of husband and wife relationship. Due to privacy and the personality of Chinese people, most respondents are unwilling to proactively expose their rapport with their spouse, affecting the final statistics.
2. Most studies have shown that emotional instability in patients can affect sexual quality of life.

After excluding those cases with depression and anxiety, they found that abnormal changes in mood can significantly affect the quality of sexual life of patients.^[17]^ Further interviews with patients showed that some patients had misunderstandings and even fears about normal sexual life, and worried that intense sexual life would increase the risk of stroke. This misunderstanding is completely unnecessary, which further confirms the lack of clinical related guidance and deserves further attention in the clinic. Therefore, we will evaluate the clinical effectiveness and safety of acupuncture in the treatment of PSED. We hope that the results of this study will provide clinicians with the basis for acupuncture treatment of PSED and more treatment options.

## 4 Trial status

At the time of manuscript submission, recruitment for the study is not yet started.

## Acknowledgments

The authors would like to thank all the trial participants. The authors are grateful for the support for this study: trial coordinating team, surgical staff, nurses, and research departments.

## Author contributions

YFL, XDY, and RJL designed the study protocol and drafted the manuscript. HSL reviewed the study protocol and drafted the manuscript. CYZ is responsible for the statistical design and analysis as trial statistician. All authors carefully read and approved the final version of the manuscript. BWL participated in the design and coordination of the study. JSW conceived and designed the study and supervised the progress of the study. All authors read and approved the final manuscript.
